# Net Reproduction Number as a Real-Time Metric of Population Reproducibility

**DOI:** 10.2196/63603

**Published:** 2025-02-12

**Authors:** Chiara Achangwa, Changhee Han, Jun-Sik Lim, Seonghui Cho, Sangbum Choi, Sukhyun Ryu

**Affiliations:** 1 Department of Preventive Medicine, College of Medicine, The Catholic University of Korea, R6117, Omibus Park, 222 Banpo-daero, Seoul, 06591, Republic of Korea, 82 02-3147-8383, 82 02-532-3820; 2 Computer Information System, Georgia State University, Atlanta, GA, United States; 3 IHAP, Université de Toulouse, INRAE, ENVT, Toulouse, France; 4 Department of Statistics, Korea University, Seoul, Republic of Korea

**Keywords:** fertility rate, reproducibility, reproduction rate, population control, Korea, sex ratio, imbalance, mortality, woman, female, childbearing age, reproductive age, giving birth, assessment, time series, Korean, impact analysis, birth control, reproduction

## Abstract

The total fertility rate (TFR) is a biased estimate of the population reproductive potential when there is a sex-ratio imbalance at birth, and it does not account for the mortality of women of childbearing age. This study aimed to estimate the reproduction rate ($R_{t}$), which adjusts for the sex-ratio imbalance and the mortality of women of childbearing age, and to assess the differences in the timing of when the population reached the replacement level of the TFR and $R_{t}$. We first estimated the $R_{t}$ using the probability of survival in women and the number of female births. Then, using a time-series analysis, we compared the time series of the TFR and $R_{t}$ in the Korean population between 1975 and 2022. We found the $R_{t}$ showed a below replacement level of the population a year earlier than the TFR. However, the estimate of the time-series analysis of $R_{t}$ was not significantly different from the estimates of the TFR. Our finding suggests that the $R_{t}$ can provide timely information on the adjusted population reproductive potential and is easier for the public to interpret compared to TFR.

## Introduction

Between 1962 and 1993, Korea implemented a successful family planning policy. In 1993, this policy was discontinued; in 2004, childbirth-promoting policies were implemented (Multimedia Appendix 1). In 2023, Korea had the lowest fertility rate (0.8) worldwide [1]. The total fertility rate (TFR), the average offspring number per childbearing-age females (15‐49 years), is a common metric to assess population change potential. However, this cohort-based measure is biased when there is male-to-female sex-ratio imbalance at birth [2,3]. The TFR does not account for mortality rates among childbearing-age women, possibly affecting population reproducibility [2,4]. These limitations reduce TFR’s ability to accurately reflect a country’s population replacement dynamics. Therefore, the net reproduction rate ($R_{t}$), the number of daughters a woman of childbearing age would produce under prevailing fertility and mortality conditions, is better. Like other real-time epidemiological metrics (eg, the effective reproduction number in infectious disease modeling) [5], the $R_{t}$ can be calculated and updated regularly with new population data; it can provide timely insights into population sustainability. The $R_{t}$ is easier for public understanding, as a population is below the replacement level when the $R_{t}$ < 1 [6], in contrast to the TFR, with a threshold of 2.1. Despite this, no previous studies have evaluated the population reproducibility using the $R_{t}$ in Korea.

This study assessed the differences in the timing of reaching population replacement level of the TFR and $R_{t}$ and the estimated difference of the time series of the TFR and $R_{t}$ by two major population control policies.

## Methods

We collected the annual number of live births, number of women, mortality rate of women, and male-to-female ratio of women of childbearing age between 1975 and 2022 through the Korean National Statistic Agency [7] to calculate the TFR and $R_{t}$ (Multimedia Appendix 2). To identify the different estimates of policy impact (1975-1993: family planning policy; 1993-2004: childbirth encouragement policy), we conducted an interrupted time series (ITS) with segmented regression to examine the time trend and its level change in the TFR and $R_{t}$. We also conducted a cross-correlation analysis to evaluate the temporal relationship between the TFR and $R_{t}$. Then, we compared the estimates of $R_{t}$ multiplied by 2.1 (TFR threshold level) with the TFR estimates along with 95% CIs. All analyses were conducted using R software (version 4.4.0; R Foundation for Statistical Computing).

## Results

**Figure 1.**
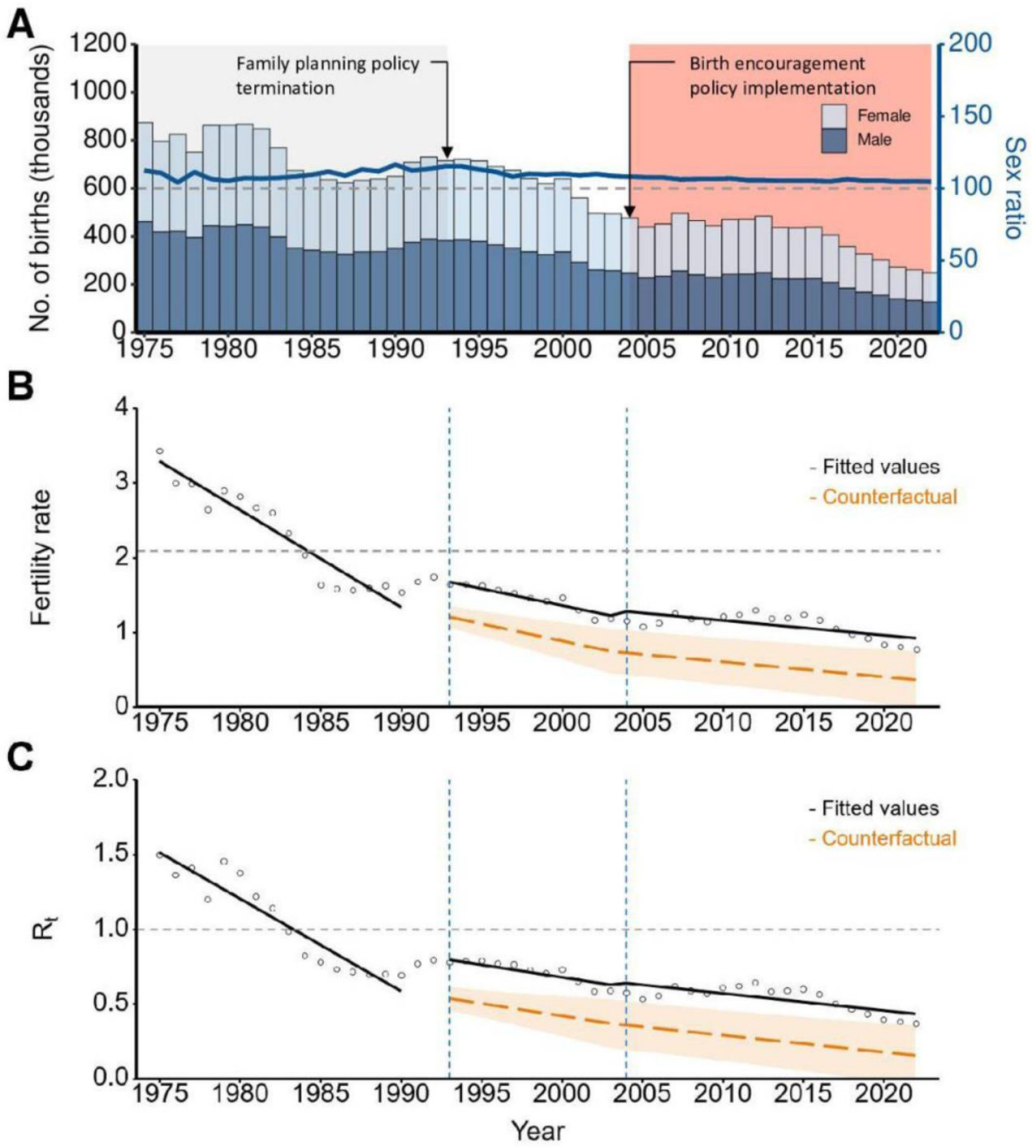
The annual number of live births, total fertility rate (TFR), and reproduction rate (R_t_) in South Korea, 1975-2022. (**A**) The bar-colored blue and sky-blue indicate the annual number of male and female births, respectively. The blue solid line indicates the yearly sex ratio of male to female births. (**B**) The interrupted time series model with the TFR. The interruption (dashed vertical line) was set to 1993 (when the family planning policy was discontinued) taking into account for the transition period of the policy and 2004 (when the birth encouragement policy was implemented) to identify the changes in the TFR level and slope. The horizontal dashed line indicates a critical threshold of the TFR at 2.1. The dashed orange line indicates the annual TFR based on a counterfactual scenario without changing the birth control policy, and the orange shaded area indicates 95% confidence intervaIs (CIs) of the TFR. (**C**) The interrupted time series model with the estimated R_t_; the critical threshold of R_t_=1. An R_t_ < 1 indicates that the population’s reproductive performance falls below the replacement level. The dashed orange line indicates the annual R_t_ based on a counterfactual scenario without changing the birth control policy, and the orange shaded area indicates 95% CIs of R_t_.

The number of live birth number decreased from 874,030 in 1975 to 249,186 in 2022. Similarly, the male-to-female sex ratio decreased from 112 in 1975 to 105 in 2022 (Figure 1A). The TFR remained below 2.1 since 1984 (TFR=2.04) and decreased further to 0.78 in 2022 (Figure 1B). The $R_{t}$ remained below 1 since 1983 ($R_{t}$=0.98) and decreased to 0.4 in 2022 (Figure 1C).

The ITS showed an immediate increase in the mean TFR (55%) and $R_{t}$ (26%) and an increased slope change of 9% in the TFR and 4% in $R_{t}$ following the family planning policy discontinuation (Figure 1B and 1C and Table 1). After the birth encouragement policy introduction, the slope of the TFR (3%) and $R_{t}$ (1%) increased, with no significant level change. When the $R_{t}$ was multiplied by 2.1, the estimates were within the 95% CI of the TFR estimate (Table 1). A high correlation between the TFR and $R_{t}$ at lag 0 indicated no temporal differences (Multimedia Appendix 3).

**Table 1.** Estimates from the interrupted time-series analysis using the total fertility rate and reproduction rate in South Korea, 1975-2022.

	Mean total fertility rate, % (95% CIs)^a^	Mean reproduction rate, % (95% CIs)^a^	Mean reproduction rate, (95% CIs) multiplied by 2.1^b^
Immediate level change following the discontinuation of family planning	54.9 (33.1 to 67.2)	25.9 (18.3 to 33.8)	54.4 (38.4 to 71.0)
Post-intervention slope change following the discontinuation of family planning	8.9 (7.6 to 10.4)	4.2 (3.3 to 5.5)	8.8 (6.9 to 11.6)
Immediate level change following the birth-encouragement policy implementation	3.2 (1.3 to 4.8)	1.2 (0.1 to 2.0)	2.5 (0.2 to 4.2)
Post-intervention slope change following the birth-encouragement policy implementation	5.8 (-5.5 to 16.4)	3.3 (-4.6 to 11.2)	6.9 (-9.7 to 23.5)

a Estimates of the mean and 95% confidence intervals (CIs) from the interrupted time series with a segmented regression model to examine the time trend and its level change.

b Estimates of the reproduction rate were multiplied by 2.1 (threshold level of total fertility rate) along with 95% CIs.

## Discussion

The threshold level of the population replacement was captured a year earlier through the $R_{t}$ compared to the TFR. This is likely due to sex-ratio imbalances in Korea.

The trend levels and slope changes of the TFR and $R_{t}$ increased following the birth control policy discontinuation [8]. These significant level changes were likely affected by previous birth control policies [9]. However, after the child encouragement policy implementation in 2004, the TFR and $R_{t}$ were far below the population replacement threshold, consistent with a previous study that reported no positive effect of child encouragement policies on the fertility rate [10], likely due to sociocultural factors influencing fertility behavior (eg, changing gender roles and economic pressures) [10]. Our study could be applied to other countries experiencing similar socioeconomic and cultural dynamics, particularly those with comparable fertility patterns and sex-ratio imbalances [4].

This study had limitations. Sensitivity analyses were not included in the parameter estimation models. The ITS models were interrupted in 1993 to reflect the discontinuation of the family planning policy, accounting for the policy transition period. The ITS may not fully capture the nonlinear trends after 2015. We did not consider the qualitative characteristics of each policy.

The $R_{t}$ can be used as a useful and timely metric of population reproducibility, particularly in the presence of sex-ratio imbalance at birth. Furthermore, the $R_{t}$ threshold of 1 may be easier for public interpretation compared to the TFR, as the public became familiar with the $R_{t}$ parameter during the COVID-19 pandemic.

## Supplementary material

10.2196/63603 Multimedia Appendix 1 Key population policies in South Korea between 1970 and 2022.

10.2196/63603 Multimedia Appendix 2 Description of total fertility rate, reproduction rate, and time-series analysis.

10.2196/63603 Multimedia Appendix 3 Yearly lagged cross-correlation coefficients between total fertility rate and net reproduction number in South Korea (1975–2022).

## Abbreviations

ITS: interrupted time series
TFR: total fertility rate

## References

[1] (2023). World population dashboard. United Nations Population Fund.

[2] Gietel-Basten S, Scherbov S (2019). Is half the world’s population really below “replacement-rate”?. PLoS ONE.

[3] Chao F, Gerland P, Cook AR, Guilmoto CZ, Alkema L (2021). Projecting sex imbalances at birth at global, regional and national levels from 2021 to 2100: scenario-based Bayesian probabilistic projections of the sex ratio at birth and missing female births based on 3.26 billion birth records. BMJ Glob Health.

[4] Aitken RJ (2022). The changing tide of human fertility. Hum Reprod.

[5] Han C, Seo H, Cho S, Chiara A, Ryu S (2023). Impact of travel restrictions for travellers from China on the internal spread of SARS-CoV-2 in South Korea. J Travel Med.

[6] Lotka AJ (1998). Analytical Theory of Biological Populations.

[7] (2023). Korean Statistical Information Service Monthly, quarterly, and annual population trends.

[8] Oh YR (2020). How does Korea’s family planning policy promote familism?. Korea Social Policy Review.

[9] Jang Y, Kim N, Lee S, Jin D (2010). Korea’s Population Policy: History and Future.

[10] Jeong K, Yoon J, Cho HJ, Kim S, Jang J (2022). The relationship between changes in the korean fertility rate and policies to encourage fertility. BMC Public Health.

