# Supplementary material for: Net Reproduction Number as a Real-Time Metric of Population Reproducibility
Source: JMIR Public Health Surveill. 2025 Feb 12;11:e63603. doi: 10.2196/63603 (PMC11837414; doi:10.2196/63603)
Supplement: Multimedia Appendix 1 [file publichealth-v11-e63603-s001.docx]

**Appendix 1.** Key population policies in South Korea between 1970-2022

| Changes in policies (effective period) | Detailed changes |
| --- | --- |
| Discontinuing the nationwide family planning strategy (1994 –) [1] | Abandoning the birth control policy, a nationwide campaign to limit the number of births and to provide financial support for vasectomy [2]. |
| Implementing child grant policy (2003 –) [3] | Providing 225-2,250 USD for two births and 375-3,750 USD for three or more births at the time of birth. The grant has varied due to the local administrative governments’ budgets [4]. |
| Implementing low birth response policy (2006 –) [5] | The policy has been implemented over four consecutive periods (2006-2010, 2011-2015, 2016-2020, and 2021-2025), supporting low-income households, promoting a work-family balance, enhancing youth employment through housing measures, and improving the quality of life for the younger generation, respectively [4, 5]. |

# **References**

1. Oh YR. How does Korea's family planning policy promote familism? Korea Social Policy Review. 2020;27(1):213-48. doi: 10.17000/kspr.27.1.202003.213.

2. Jang Y, Kim N, Lee S, Jin D. Korea's Population Policy: History and Future. 2010.

3. Suk H-W. Study on the impact of Maternity subsidies policy: Focusing on local governments in Seoul Metropolis. Local administration research. 2011;25(2):143-80.

4. Lee B-H, Park M-G. Determinants of Third or Higher Births in Korea: Focusing on the Effects of Cash Grants. Health and Social Welfare Review. 2017;37(3):318-42. doi: DOI :10.15709/hswr.2017.37.3.318.

5. National Assembly Budget Office. Analysis and evaluation of low birth rate response projects. Seoul, Korea: National Assembly Budget Office, 2021 23 August 2021.
