# Supplementary material for: Net Reproduction Number as a Real-Time Metric of Population Reproducibility
Source: JMIR Public Health Surveill. 2025 Feb 12;11:e63603. doi: 10.2196/63603 (PMC11837414; doi:10.2196/63603)
Supplement: Multimedia Appendix 2 [file publichealth-v11-e63603-s002.docx]

**Appendix 2.** Description of total fertility rate, reproduction rate, and time-series analysis

|  | Description |
| --- | --- |
| Total fertility rate | We calculated the annual total fatality rate (TFR), defined as the average number of offspring per all females of childbearing age (i.e., 15-49 years), using the number of live births and number of women of childbearing age. |
| Reproduction rate | We estimated the net reproduction rate ($R_{t}$) to measure the reproductive performance (i.e., reproducibility of the population) in South Korea. $R_{t}$ represents the mean number of female offspring per woman of childbearing age (i.e., 15-49 years) in the year *t*. An $R_{t}$ < 1 indicates that the population reproducibility fell below the replacement level.[1-3] We first obtained the annual number of live births and the male-to-female ratio from women of childbearing age between 1970 and 2022 through the Korean national statistics authorities to calculate the mean number of female births.[4] We also collected the population number with the mortality rate of females aged 15-49 to estimate the probability of survival in women of childbearing age. Then, we estimated the annual reproduction rate between 1970-2022 as follows:  $Reproduction rate \left( R_{t} \right)= \sum_{i=15}^{49} S_{i}B_{i}$,  where $S_{i}$ denotes the probability of survival in females aged 15-49 and $B_{i}$ denotes the mean number of female births from a woman aged 15-49. |
| Time series analysis | We conducted an interrupted time series with segmented regression to assess the time trend and level change in the reproduction rate (log($R_{t}$)) of the South Korean population. We used linear regression, including the population control policies of discontinuing family planning and encouraging childbirth, and the regression coefficients to estimate the pre-intervention slope, immediate level change following the policy implementation, and changes in the post-intervention slope of $log(R_{t})$. The model included variables to indicate the pre- or post-intervention period for each policy. We applied backward selection using the Chi-square test to exclude variables that were not significantly related to $log(R_{t})$. Mathematically, the model is as follows:  $Log\left( R_{t} \right)=\beta_{0}+\beta_{1}\times{year}_{t}+\beta_{2}\times{discontinuing family planning policy}_{t}+\beta_{3}\times year after {discontinuing family planning policy}_{t}+\beta_{4}\times{birth encouragement policy}_{t}+\beta_{5}\times year after {birth encouragement policy}_{t}+ e_{t}$  where $\beta_{0}$ estimates the baseline level of $Log\left( R_{t} \right)$; $\beta_{1}$ estimates the baseline trend of $Log\left( R_{t} \right)$; $\beta_{2}$ estimates the level change following the discontinuation of the family planning policy; $\beta_{3}$ estimates the trend change after the discontinuation of the family planning policy; $\beta_{4}$ estimates the level change following the birth encouragement policy; and $\beta_{5}$ estimates the trend change after the birth encouragement policy. |

**References**

1. Alfred J. Lotka. Analytical Theory of Biological Populations. New York, NY: Springer New York, NY; 1998. ISBN: 978-0-306-45927-6.

2. Birley MH. The Estimation of the Net Reproductive Rate (R0) of Multivoltine Pest Populations from Census Data. Journal of Animal Ecology. 1978;47(3):689-96. doi: https://doi.org/10.2307/3664.

3. United Nations Population Fund. World Population Dashboard. New York, United States: UNFPA; 2023 [cited 2 January 2024]; Available from: https://www.unfpa.org/data/world-population-dashboard.

4. Korean Statistical Information Service. Monthly, quarterly, and annual population trends. Daejeon, South Korea: Statistics Korea; 2023 [cited 2 January 2024]; Available from: https://kosis.kr/statHtml/statHtml.do?orgId=101&tblId=DT_1B8000G&conn_path=I2.
