# Supplementary material for: Net Reproduction Number as a Real-Time Metric of Population Reproducibility
Source: JMIR Public Health Surveill. 2025 Feb 12;11:e63603. doi: 10.2196/63603 (PMC11837414; doi:10.2196/63603)

**Appendix 3.** Yearly lagged cross-correlation coefficients between total fertility rate and net reproduction number in South Korea (1975–2022). The dark vertical solid lines represent the correlation coefficients for each lag, while the dashed horizontal blue lines indicate the thresholds beyond which the correlations are considered statistically significant


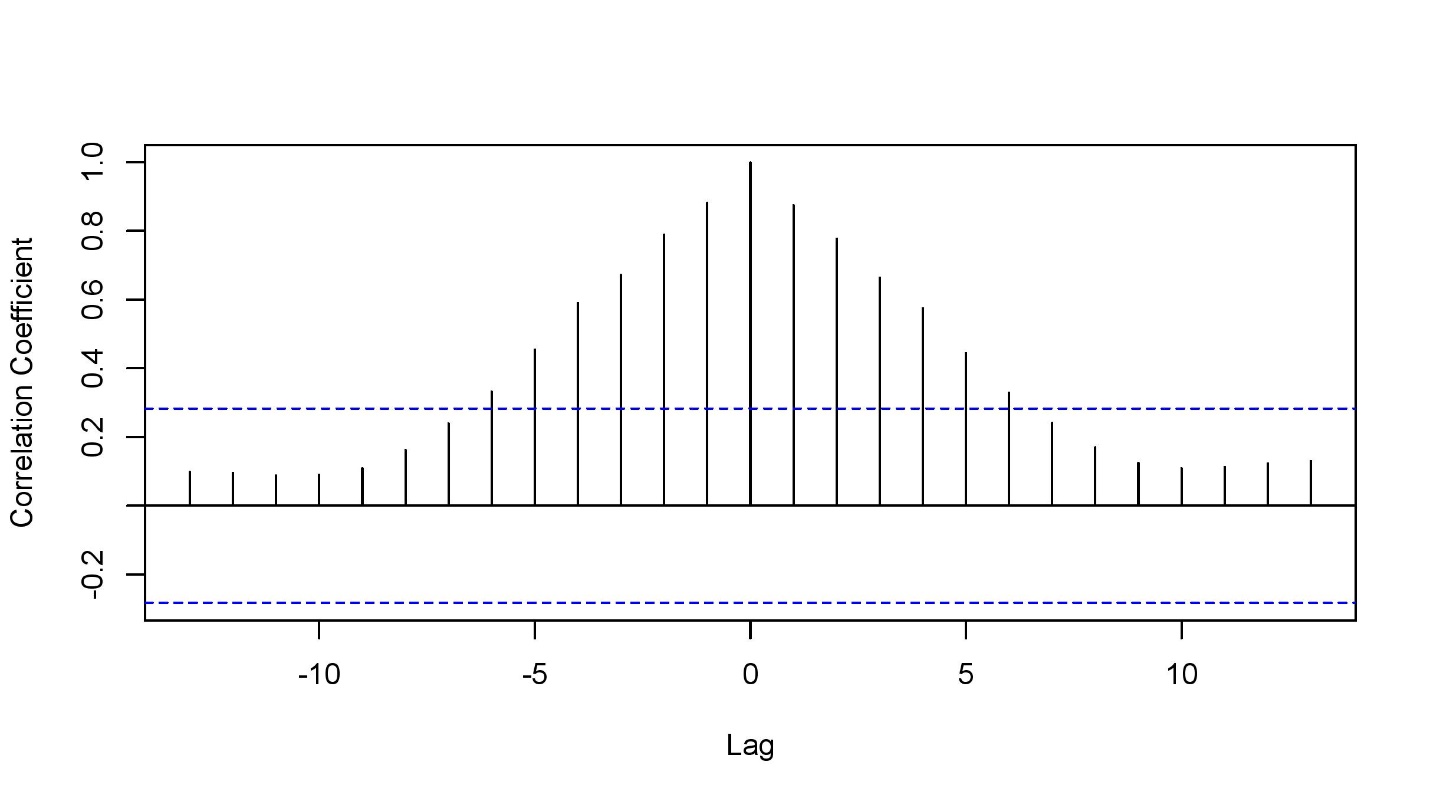

Supplement: Multimedia Appendix 3 [file publichealth-v11-e63603-s003.docx]
